# Lung Cancer Imaging: Screening Result and Nodule Management

**DOI:** 10.3390/ijerph19042460

**Published:** 2022-02-21

**Authors:** Susanna Guerrini, Davide Del Roscio, Matteo Zanoni, Paolo Cameli, Elena Bargagli, Luca Volterrani, Maria Antonietta Mazzei, Luca Luzzi

**Affiliations:** 1 Unit of Diagnostic Imaging, Department of Radiological Sciences, Azienda Ospedaliero-Universitaria Senese, University of Siena, 53100 Siena, Italy; 2 Unit of Diagnostic Imaging, Department of Medical, Surgical and Neuro Sciences and of Radiological Sciences, Azienda Ospedaliero-Universitaria Senese, University of Siena, 53100 Siena, Italy; davide.delroscio90@gmail.com (D.D.R.); matteo.zanoni.91@gmail.com (M.Z.); luca.volterrani@unisi.it (L.V.); mariaantonietta.mazzei@unisi.it (M.A.M.); 3 Respiratory Diseases Unit, Department of Medical, Surgical and Neuro Sciences, Azienda Ospedaliero-Universitaria Senese, University of Siena, 53100 Siena, Italy; cameli3@student.unisi.it (P.C.); bargagli2@unisi.it (E.B.); 4 Italian Society of Medical and Interventional Radiology (SIRM), SIRM Foundation, 20122 Milan, Italy; 5 Lung Transplant Unit, Department of Medical, Surgical and Neuro Sciences, Azienda Ospedaliero-Universitaria Senese, University of Siena, 53100 Siena, Italy; dr.luca.luzzi@gmail.com

**Keywords:** lung cancer, prevention, health education, MDCT, lung screening, lung nodule management

## Abstract

*Background***:** Lung cancer (LC) represents the main cause of cancer-related deaths worldwide, especially because the majority of patients present with an advanced stage of the disease at the time of diagnosis. This systematic review describes the evidence behind screening results and the current guidelines available to manage lung nodules. *Methods:* This review was guided by the Preferred Reporting Items for Systematic Reviews and Meta-analyses (PRISMA) guidelines. The following electronic databases were searched: PubMed, EMBASE, and the Web of Science. *Results:* Five studies were included in the systematic review. The study cohort included 46,364 patients, and, in this case series, LC was detected in 9028 patients. Among the patients with detected LC, 1261 died of lung cancer, 3153 died of other types of cancers and 4614 died of other causes. *Conclusions:* This systematic review validates the use of CT in LC screening follow-ups, and bids for future integration and implementation of nodule management protocols to improve LC screening, avoid missed cancers and to reduce the number of unnecessary investigations.

## 1. Introduction

Lung cancer (LC) represents the main cause of cancer-related deaths worldwide, especially because the majority of patients present with an advanced stage of the disease at the time of diagnosis [1,2]. Smoking habits and asbestos exposure are still the main risk factors; moreover, smoking prevalence is going to rise in the coming years [3,4,5,6,7]. For these reasons, mass screening of high-risk patients has been introduced in most countries, with the aim of reducing LC mortality. This seems to be potentially beneficial; however, the frequent identification of early stage cancers could lead to the over diagnosis and unnecessary treatment of early lesions that would potentially never become advanced cancers [8]. To address this issue, in Europe and in the US, many multicenter trials were instituted [9,10,11,12,13] to investigate whether screening using multidetector computed tomography (MDCT) would contribute to the reduction in LC mortality [14]. MDCT, with its widespread use and the possibility to use low-dose CT (LDCT), dual-energy CT (DECT) and functional acquisitions, such as CT perfusion (CTp), allows the detection and characterization of early stage LC [15,16,17,18].

Lung cancer can present as solid, part-solid or ground-glass nodules [19,20]. Sometimes, LC grows near cystic airspaces, as thickening of the wall of a bulla, or present as a mass at the first diagnosis [21]. New-generation CT scanners allow the detection of pulmonary lesions, not absolutely malignant, with a prevalence of about 50%, which imposes a tight follow-up, both in screening and diagnostic protocols [22].

For these reasons, nodule management guidelines are necessary to advise on how to manage early detected lesions that need close follow-ups, to correctly identify lesions with a high malignant potential, and to reduce the number of unnecessary examinations or surgical excisions [19].

The purpose of screening is to detect treatable lesions before they become clinically evident, and to address the management of pre-invasive nodular lesions, thereby reducing patient mortality and over-treatment [23].

The aim of this systematic review is to describe the evidence behind screening results and the current guidelines available to manage lung nodules, as well as the indications and appropriate timing of surveillance.

## 2. Materials and Methods

### 2.1. Publication Search

This systematic review was conducted according to the Preferred Reporting Items for Systematic Reviews and Meta-analyses (PRISMA) guidelines.

To find studies to include in this systematic review, we searched the PubMed, EMBASE, and Web of Science electronic databases for articles, using the terms “lung cancer”, “lung carcinoma”, “imaging”, “solitary pulmonary nodules”, “lung nodule management”, “pulmonary screening”, “lung nodules”, “computed tomography”, “lung cancer screening result”, and “lung cancer guidelines”. We scrutinized studies published from January 2010 to January 2020, which had been published in English and had been conducted on humans. We also performed manual retrieval to avoid missing important studies. Only full-text articles were included.

### 2.2. Inclusion and Exclusion Criteria

Inclusion criteria were as follows: (1) LC histopathologically or cytologically proven, (2) CT image confirming lung lesion.

Studies were excluded from the analysis for (1) lack of CT imaging or histopathological/cytological results confirming LC; (2) cases of duplicate or overlapping data with other studies; (3) articles were abstracts, case reports, comments; (4) English full-text article was not available.

We evaluated the title and the abstract when the title suggested that the study met the inclusion criteria. If the abstract was relevant, the articles’ full versions were read by two of the authors. Disagreements were resolved by a combined full article review and by consensus between the authors.

### 2.3. Data Extraction

Data were extracted independently by two co-authors and discrepancies were resolved by consensus. The following information was considered valid for the analysis: year of publication, country of origin, the number of included patients, age, sex, cause of death and smoking status.

Simultaneously, recent guidelines for monitoring lung nodules to compare nodule timing of surveillance, according to frequent imaging findings, were evaluated, including the following: ground-glass (GG) nodules, solid nodules, and part-solid (PS) nodules. Nodule multiplicity, growth rate, location, size and morphology were also evaluated.

## 3. Results

### 3.1. Included Studies

**Figure 1:**
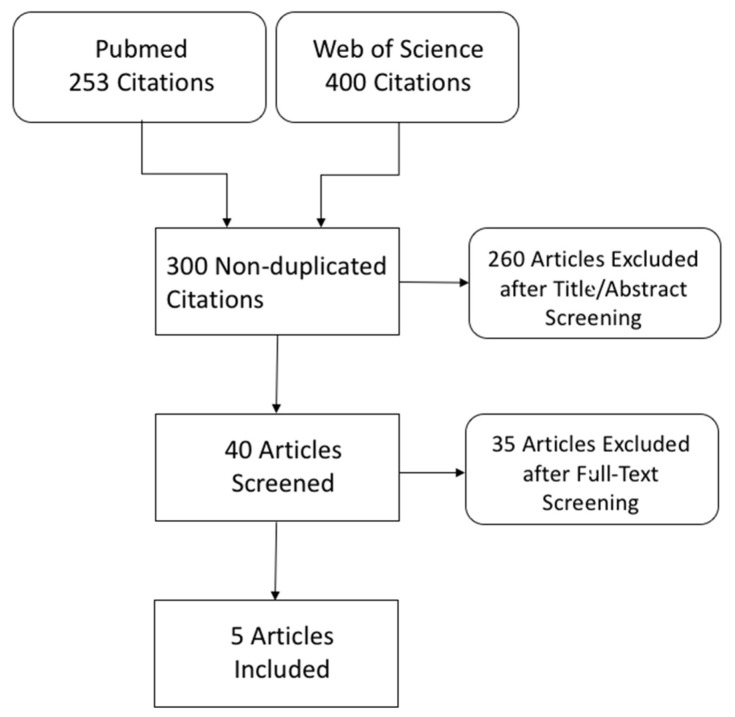
Flowchart of the literature search and study selection.

Our literature search yielded 653 citations. Forty non-duplicated records were eligible for full-text screening, and were examined in detail. Five studies were included in the screening study. A flowchart of the literature search and study selection is shown in Figure 1, and the results are summarized in Table 1.

For lung nodule guidelines, the evaluation of four articles was included [19,24,25,26]. All the articles report predictors of malignancy, nodule morphology and size. Comparisons between the guideline recommendations for nodule management are reported in Table 2 and Table 3.

### 3.2. Patient Characteristics for Screening Results

The study cohort included 46,364 patients. In this case series, LC was detected in 9028 patients. The following clinical stages were reported in 1865 patients in total: stage IA (647, 34.69%), stage IB (174, 9.32%), stage II (262, 14.04%), stage III (334, 17.90%), stage IV (345, 18.49%), and unknown (103, 5.52%). A total of 149/1865 (7.98%) patients had NSCLC, 844/1865 (45.25%) had adenocarcinoma, 404/1865 (21.66%) had squamous cell carcinoma, 133/1865 (7.13%) had small-cell carcinoma, and 335/1865 (17.96%) had another type of lung cancer (unknown). Among the patients with detected LC, in a follow-up period ranging from 5 to 10 years, 1261 patients died of lung cancer, 3153 patients died of other types of cancer, and 4614 patients died of other causes.

## 4. Discussion

**Figure 2:**
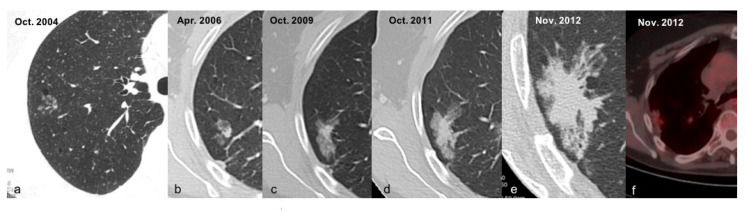
(**a**–**f**) This is an example of a PS pre-malignant nodule (**a**) with evolution in malignant lesion (**a**–**e**), confirmed by PET-CT (**f**), in accordance with changes in the solid component of the nodule.

All the evaluated studies agree with the importance of CT/LDCT screening in reducing LC mortality, which was reduced by a percentage of approximatively 20% in a screening follow-up period of about 10 years. In the de Koning et al. trial, involving high-risk patients (current smokers), there was an increase in LC diagnoses among patients who underwent CT screening when compared with patients who did not adhere to the screening program [13]. In the National Lung Screening Trial (NLST) by Aberle et al., the reduction in mortality of patients who underwent LDCT screening was assessed in comparison with radiography. Even though LDCT reported a high rate of false positive results, it does not seem to be deleterious, considering the decrease in the LC mortality rate [11]. In the DANTE trial (Infante et al., 2015), the false positives and stage I lung cancer rates are comparable with NLST; however, the authors imposed a lower age limit of 60 years to maximize the efficiency of screening [9] compared to other randomized LDCT screening trials [28,29,30]. Infante et al. (2017) reported an overall mortality reduction of 11% at the 8-year follow-up, which is no different from the NLST trial, if compared with the small number of participants, and with an increased reduction after the 4-year follow-up [12]. In Horeweg et al., the authors evaluated the characteristics of screening-detected LC in the NELSON trial, encouraging a regimen of LC screening of 2 years, with high specificity and sensitivity [10]. The detection of pulmonary nodules during screening in asymptomatic patients increases the number of early detected LC, and also the number of undefined lung nodules that require surveillance during the patient’s lifetime. A correct screening algorithm and classification of detected nodules are essential, even though the best management protocol to define malignant versus benign lesions remains under debate [31]. MDCT screening represents a path towards the resolution of this issue; however, there are many differences that exist between the guidelines for lung nodule management [19,24,25,26]. Even though there is substantial concordance in all trials, regarding the management of solid nodules, especially for nodules <6 mm that require optional follow-up, greater lesions (>8 mm) should be managed in accordance with the nodule morphology and modifications during the follow-up period, making CT the most suitable surveillance method [24]. In de Koning et al., annual LDCT screening was recommended for people at high risk of LC by the US Preventive Services Task Force and medical societies, according to the American College of Chest Physicians guidelines [13,24]; however, no reduction in LC mortality with the use of the same guidelines has been reported in Infante et al. (2015) [9], possibly due to its smaller sample sizes. For this reason, in 2017, Infante et al. conducted a patient-level pooled analysis of two Italian randomized controlled trials [12]; however, a nonsignificant (11%) reduction in overall mortality in individuals undergoing LDCT screening was found. Horewag et al. and Aberle et al. [10,11] adhered to the same guidelines, achieving similar results to de Koning et al., showing a decrease in death rate with the use of LDCT in LC screening [13]. PS and GG nodule management are the most challenging; PS lesions are often pre-malignant or malignant, in accordance with the evolution of the solid component of the nodule, and, for this reason, they require closer surveillance [24] (Figure 2a–f).

**Figure 3:**
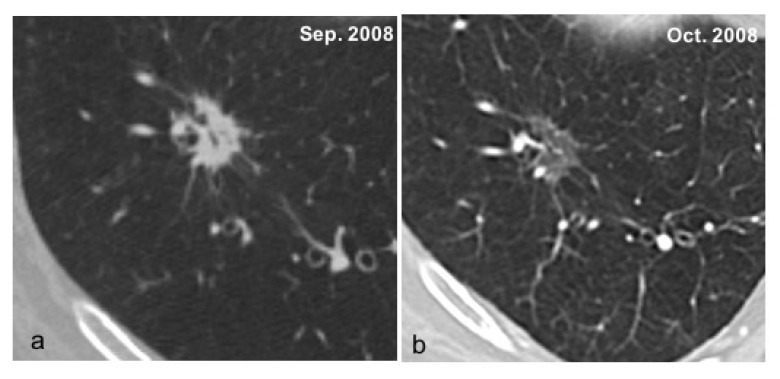
(**a**,**b**) This is an example of a solid nodule (**a**) suspected for malignant lesion. However, at 1-month follow-up CT examination, it tends to disappear due to its inflammatory nature (**b**).

GG lesions were considered possibly malignant if they were larger than 30 mm, or in the case of persistence during follow-up. The greatest number of GG lesions <6 mm tended to disappear by the 6-month follow-up CT examination, due to their inflammatory nature (bronchiolitis, etc.) (Figure 3a,b) [18].

However, nodule measurements can be affected by different biases, related to the irregular shape, closeness to other structures (blood vessels, bronchi, etc.), motion artefacts (related to the patient or due to the proximity with the heart), or superimposed diseases that partially or totally cover the nodule [25]. No considerations were reported about GG or PS lesion detection and characterization in all the evaluated trials.

**Figure 4:**
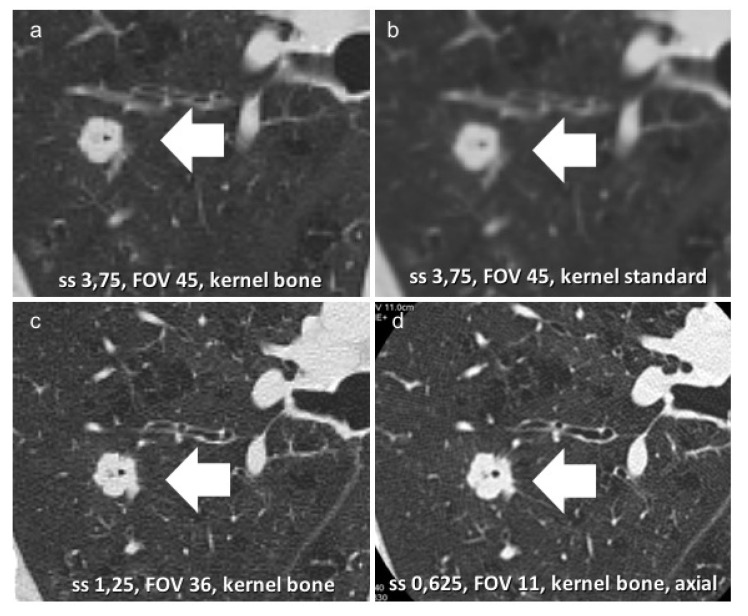
(**a**–**d**) This is an example of a solid nodule suspected for malignant lesion (**a**–**d**). Reconstruction using a high spatial frequency algorithm (bone plus, (**a**,**c**,**d**)) with a thinner acquisition (0.625 mm, axial, (**d**)), which allows the characterization of this malignant lesion.

Concerning technical acquisition protocols, some considerations are necessary. Many studies demonstrate non-significant differences in the variability of the measured nodules between LDCT and standard-dose CT (also not affecting nodule detection) if a maximum section thickness of 1.25 mm was reported and contiguous section CT images were acquired (evidence level 2+) [32,33,34,35]. In the literature search, no study reported any difference between LDCT and standard-dose CT for pure GG nodule characterization; this is because most studies were aimed at nodule detection, even though slice thickness can affect nodule morphological evaluation. A thinner acquisition or reconstruction using a high spatial frequency algorithm (bone plus), with 1.25 mm slice thickness, allows the detection of “signs of malignity” (bronchus sign, pleural tag, and spiculate shape), which are highly suggestive of invasive lesions, regardless of the dimensions and growth timing [36,37] (Figure 4a–d).

**Figure 5:**
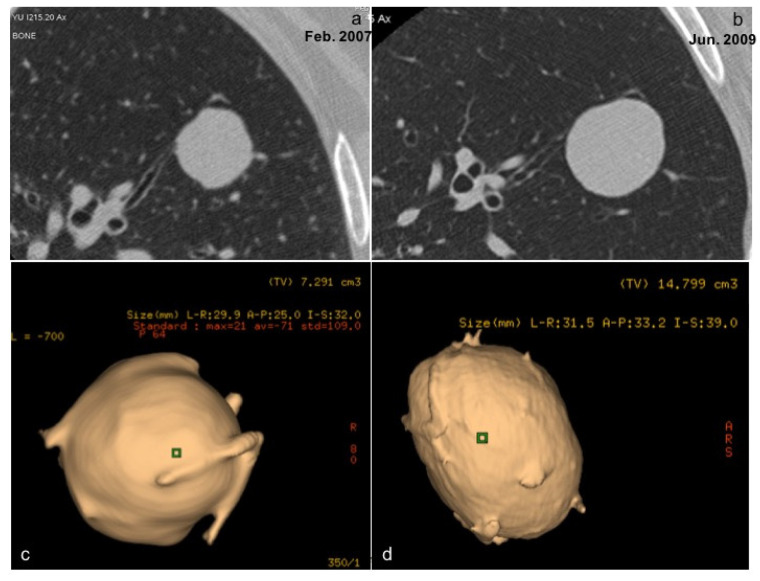
(**a**–**d**). This is an example of a solid nodule, amartocondroma (**a**,**c**), with a fast growth (2 years), confirmed with 3D reconstructions (**b**,**d**).

However, most LDCTs use a section thickness of 2.5 mm to assess for interval changes in size and density, and to detect and measure solid components, which are sufficient to address nodule management [38]. The volume doubling time (VDT) remains the most attractive option in lung nodule follow-up; however, many issues influence this choice, such as the non-linear growth of some lesions, rapid spread of adenocarcinomas from pre-invasive lesions, and the possibility of benign lesions to grow quickly [39,40,41] (Figure 5a–d).

Artificial intelligence (AI) should also be mentioned, which, through automated detection, segmentation, and computer-aided diagnosis (CAD), helps in the identification of suspicious lesions [42]. Radiomics represents a promising AI algorithm, based on the extraction of a data set of features from an image, which allows for the automated classification of medical images. The features evaluated mainly concern nodule shape; however, at the time of writing this paper, more than 100 features have been defined [42,43,44,45]. Unfortunately, radiomic signatures are not standardized yet, and the lack of optimal reproducibility makes radiomics impossible to use in screening programs; though, in the future, it may represent a valid tool for LC detection. Similarly, CTp can be used to differentiate benign from malignant lesions throughout the functional imaging information provided by imaging of the first pass of the contrast medium through the interested tissue [46]. In particular, CTp can be used to distinguish LC from inflammatory nodules on the basis of the permeability surface (PS), derived from CTp acquisitions. However, CTp has not been routinely utilized in clinical practice, despite the wide distribution of commercial CTp software [47].

## 5. Conclusions

This systematic review arises from a vast and meticulous research of the literature regarding screening results and lung nodule management. Many articles were published between 2010 and 2020 because the technological evolution and widespread diffusion of MDCT produced rapid changes in lung nodule management. The results highlight the efficacy of the American College of Chest Physicians guidelines for lung nodule management in decreasing the rate of deaths when using LDCT for LC screening. The results remain uncertain regarding long-term follow-up; it is, however, possible that a longer follow-up time (>10 years), especially for pure GG nodules, could lead to advantages that should be further investigated. On the other hand, a unique 3-month follow-up CT scan seems to be effective in assessing the resolution of infected nodules. Future integration and implementation of nodule management protocols is desirable to achieve an improvement in screening for LC, to avoid missed cancers and to reduce the number of unnecessary investigations.

## Figures and Tables

**Table 1:** Screening study results.

	De Koning 2020 [13]	Infante 2017 [12]	Infante 2015 [9]	Horeweg 2014 [10]	Aberle 2011 [11]
Mean Age	58 yo	61 yo	64 yo	58 yo	NR
Male Sex	All	2890	NR	5999	15,770
Smoking status *	All	2344	714	3959	12,862
Mean p/y **	38	40	47.3	38	NR
Patients	6583	3640	1264	7155	27,722

* Current smoker was defined as a person who had smoked cigarettes during the last 2 weeks. ** Calculated by multiplying the number of packs of cigarettes smoked per day by the number of years the person had smoked. Yo = year old; NR = not reported. The studies come from different countries, the largest from the United States [11,13] and others from Europe, including Italy [9,12] and The Netherlands [10]. All articles are randomized trials, 3 with a control arm.

**Table 2:** Comparison of current guidelines for solid nodule management [27].

Nodule Dimensions	The Fleischner Society [19]	American College of Chest Physicians [24]	British Thoracic Society [25]	Lung CT Screening Reporting and Data System * [26]
<6 mm	LR, no FU HR, 12 mo FU	LR, ≤4 mm no FU LR, >4–6 mm or HR, ≤4 mm, 12 mo FUHR, >4–6 mm, 6–12 mo FU	<5 mm, no FU5–6 mm, 12–24 mo FU	<6 mm, AS (cat 2)
≥6 mm to 8 mm	LR and HR, 6–12 mo FU, then re-evaluate	LR, 6–12 mo FU HR, 3–6 mo FU	3 mo FU then 12 mo FU	≥6 mm or new nodules 4–6 mm, 6 mo LDCT (cat 3)
≥8 mm	CT or PET/CT at 3 mo	<5% risk, 3 mo FU; 5–65% risk, PET/CT and/or biopsy; >65% risk, treatment	<10% risk, surveillance; >10% risk, PET/CT or consider resection	8–15 mm, 3 mo LDCT (cat 4A) >15 mm (cat 4B)

* Lung-RADS^®^ version 1.1; assessment categories release date: 2019. High-risk factors include older age, heavy smoking, larger nodule size, irregular or spiculated margins, and upper lobe location. HR = high risk; LR = low risk; mo = months; FU = follow-up; AS = annual screening; cat = category; LDCT = low-dose computed tomography.

**Table 3:** Comparison of current guidelines for ground-glass and part-solid nodule management [27].

Nodule Dimensions	The Fleischner Society [19]	American College of Chest Physicians [24]	British Thoracic Society [25]	Lung CT Screening Reporting and Data System * [26]
<6 mm	<6 mm, GG or PS, no FU; if multiple, 3–6 mo LDCT FU	<6 mm, GG, no FU	<5 mm, no FU	30 mm or more, GG, AS (cat 2) 6 mm, PS, AS (cat 2) if new 6 mo LDCT (cat 3)
≥6 mm to 8 mm	≥6 mm, GG, 6–12 mo FU; PS, 3–6 mo FUIf multiple, 3–6 mo FU	≥6 mm GG, 12 mo FU; PS, ≤8 mm, 3, 12, and 24 mo FU	≥5 mm, 3-mo LDCT than re-evaluate	≥30 mm, GG or new 6 mo LDCT (cat 3) 6–8 mm, PS, 3 mo LDCT (cat 4A)
≥8 mm	/	If solid, 3 mo FU	/	≥8 mm, PS, (cat 4B)

* Lung-RADS^®^ version 1.1; assessment categories release date: 2019. GG = ground glass; PS = part solid; mo = months; FU = follow-up; AS = annual screening; cat = category; LDCT = low-dose computed tomography.
